# Τhe Greek Version of the Marwit–Meuser Caregiver Grief Inventory (MM-CGI) and MM-CGI Short Form (MM-CGI-SF): An Examination of Their Psychometric Properties in Family Caregivers of Persons with Dementia Before Death

**DOI:** 10.3390/bs15020219

**Published:** 2025-02-15

**Authors:** Efthymia Tsigkou, Konstantina Athina Tsironi, Georgia Papantoniou, Maria Sofologi, Georgios Kougioumtzis, Ioanna Giannoula Katsouri, Despina Moraitou, Magda Tsolaki

**Affiliations:** 1 Neurosciences and Neurodegenerative Diseases Postgraduate Course, Medical School, Faculty of Health Sciences, Aristotle University of Thessaloniki, 54124 Thessaloniki, Greece; 2 Department of Caregiver’s Support, Greek Association of Alzheimer’s Disease and Related Disorders (GAADRD), 54643 Thessaloniki, Greece; konath.tsironi@gmail.com (K.A.T.); tsolakim1@gmail.com (M.T.); 3 Laboratory of Psychology, Department of Early Childhood Education, School of Education, University of Ioannina, 45110 Ioannina, Greece; gpapanto@uoi.gr (G.P.); m.sofologi@uoi.gr (M.S.); 4 Laboratory of Neurodegenerative Diseases, Center for Interdisciplinary Research and Innovation (CIRI-AUTH), Balkan Center, Aristotle University of Thessaloniki, 10th km Thessaloniki-Thermi, 54124 Thessaloniki, Greece; demorait@psy.auth.gr; 5 Department of Turkish Studies and Modern Asian Studies, Faculty of Economic and Political Sciences, National Kapodistrian University of Athens, 15772 Athens, Greece; gkougioum@ppp.uoa.gr; 6 Department of Psychology, School of Health Sciences, Neapolis University Pafos, 8042 Pafos, Cyprus; 7 Department of Occupational Therapy, University of West Attica, 12241 Athens, Greece; ykatsouri@uniwa.gr; 8 Laboratory of Psychology, Section of Cognition, Brain and Behavior, School of Psychology, Aristotle University of Thessaloniki, 54124 Thessaloniki, Greece

**Keywords:** caregiver’s grief, dementia, family caregivers, psychometric properties

## Abstract

Family caregivers of dementia patients experience grief, not only after the death of the patient, but also during the course of the disease. The aim of the present study was to examine the psychometric properties of the Greek version (full and short) of the Marwit–Meuser Caregiver Grief Inventory (MM-CGI and MM-CGI Short Form) before death in a sample of Greek caregivers of persons with dementia. In particular, the study attempts to test the internal consistency reliability as well as the structural and convergent validity of the inventory. The inventory was administered to 515 family caregivers (offsprings and spouses) along with a socio-demographic information form, the Zarit Burden Interview (ZBI), the Beck Depression Inventory II (BDI-II), and the Mini-Mental State Examination (MMSE) exploratively to a subset of dementia patients cared for by survey participants. The application of confirmatory factor analyses revealed a slightly different structure of the Greek version of the MM-CGI, but they fully verified the structure of the Greek version of the MM-CGI-SF. Both the internal consistency reliability and the convergent validity of the subscales of the Greek version of the MM-CGI-SF were good. The findings of the present study indicate that the short version of the Marwit–Meuser Caregiver Grief Inventory is a reliable and valid tool for the early assessment of aspects of grief among the Greek population of family caregivers of dementia patients, in order to potentiate the prevention of their depression and anxiety.

## 1. Introduction

The grief of family caregivers, which is an irremovable part of the whole experience of caregiving, is often overlooked. Quite recently, the scientific community shed light on the examination of this type of grief, which is called “anticipatory grief”, “pre-death grief”, or simply grief of caregivers. The grief of caregivers is considered by many researchers as a principal component of the experience of caregiving, much like the burden, depression, and anxiety of caregivers. Thus, the importance of evaluating this part of the experience of caregivers is vital, especially for family caregivers who already have an emotional bond with their patient. For example, caregivers who are spouses of dementia patients experience high levels of grief, either normal or complicated (D. Chan et al., 2013; Marwit & Meuser, 2002; Meuser & Marwit, 2001).

Grief in dementia caregivers may be normal or complicated and can be experienced before and after death (D. Chan et al., 2013). Grief as a reaction to the perception of loss “with symptoms unique to the individual” (Rando, 2000) can be expected. Anticipatory grief can be experienced in the whole course of the disease, but it is more present at moderate and severe stages of dementia (Holley & Mast, 2009). However, high levels of anticipatory and complicated grief put caregivers in an at-risk population. For instance, it has been reported that the depression of dementia caregivers increases with anticipatory grief (D. Chan et al., 2013). According to the review of D. Chan et al. (2013), anticipatory grief percentages ranged between 47% and 71%, whereas complicated grief percentages after the death of the patient were estimated around 20%.

The nature of the neurodegenerative disease of dementia causes a peculiar experience of grief with special characteristics in family caregivers of persons with dementia. First of all, family caregivers experience losses not only after the death of their patient with dementia but also during the life of the patient (D. Chan et al., 2013). Secondly, the population of caregivers of persons with dementia not only faces the impending death, but also the grief of changes that have already occurred and the losses of the future while dementia progresses (Rando, 1986). In addition, family caregivers of this population experience losses on many different levels, such as on a relational level, on a personal level, and on a social life level (D. Chan et al., 2013; Meuser & Marwit, 2001). Finally, caregivers experience continuous stepwise losses. We could call the whole experience of caregivers’ grief as an “*affective marathon*”.

In Greece, the institution of the family is strong. The undertaking of the role of caregiver is a common response for spouses and offsprings of persons with dementia. There is also a deep-rooted culture of providing care at home as much as possible, in order to offer the warmth and comfort of the family environment. This means that the family caregivers in Greece experience the progressive disease of dementia up close and with a demanding commitment to the role. This may result in higher reactions in terms of sentiments of loss of the relationship with the patient (e.g., changes in everyday life, in the family roles and the interpersonal relationships, communication disorder) and in scenarios in which the responsibilities to the person with dementia provoke losses in the personal life of the caregiver (e.g., loss of personal freedom, abandonment of social or work life) (Bowd, 1997; Farran et al., 1991; Sanders & Corley, 2003). Papastavrou and her colleagues found that a significant percentage of caregivers experience a high burden and manifest symptoms of depression (Papastavrou et al., 2007). A cross-sectional study in Greece concerning the caregivers of persons with mental disorders showed that the mentioned population suffers from stress, anxiety, and depression of significant degree, with an impact on their quality of life (Oikonomou et al., 2024).

This context highlights the critical need to focus on pre-death grief as well as on burden, anxiety, depression, and the post-death stage of the family caregiver.

The preparedness of a dementia family caregiver for the impending death of the patient could improve the experience of grief (Hebert et al., 2006; Nielsen et al., 2016). It is more possible for caregivers who are not prepared for the impending death to develop depression and anxiety during the grief after the death of the patient (Holley & Mast, 2009). Furthermore, it is already known that grief is a risk factor for the emergence of depression and anxiety (Cole & Dendukuri, 2003). Thus, the evaluation of pre-death grief could be a first step in order to intervene promptly and protect the mental health of family caregivers.

The first scale of the assessment of anticipatory grief of dementia caregivers was created by Theut and her colleagues in 1991 (Theut et al., 1991). The inventory of grief of family caregivers of patients with dementia by Marwit and Meuser (Marwit–Meuser Caregiver Grief Inventory—MM-CGI) followed in 2002, and the short form of the MM-CGI (Marwit–Meuser Caregiver Grief Inventory Short Form—MM-CGI-SF) was created by the same researchers in 2005 (Marwit & Meuser, 2002, 2005). According to a recent systematic review in respect of the assessment of anticipatory grief of dementia caregivers (Dehpour & Koffman, 2023), psychometric properties of the MM-CGI and MM-CGI-SF have been examined in studies since 2005.

The MM-CGI and MM-CGI-SF are considered hallmarks of the investigation of grief of family caregivers of persons with dementia. The MM-CGI inventory is a self-report questionnaire with 50 items, and it is designed to measure the reactions of grief experienced by current family caregivers, spouses and offspring, of persons with dementia (Marwit & Meuser, 2002). The inventory is based on the model of grief of caregivers and clinical assessment of Marwit and Meuser (Meuser & Marwit, 2001). The MM-CGI has three factors (subscales) representing three different domains of the grief of family caregivers:

Factor 1: Personal Sacrifice Burden (PSB)
Factor 2: Heartfelt Sadness and Longing (HSL)
Factor 3: Worry and Felt Isolation (WFI)

The three factors were developed via the multidimensional model of a research work of Marwit and Meuser based on the caregivers’ grief for persons with dementia in 2001 (Meuser & Marwit, 2001). Factor 1 has 18 items, Factor 2 has 15 items, and Factor 3 has 17 items. In respect of internal consistency, the items of the MM-CGI inventory, as well as of each of its three subscales, showed excellent reliability coefficients (Cronbach’s *α* of 0.96 for the total inventory, and of 0.93, 0.90, and 0.91, respectively, for each subscale) (Marwit & Meuser, 2002).

The MM-CGI received encouraging feedback from the scientific community, but Marwit and Meuser commented that “a short-form would be welcomed for use in situations where time is a factor, either because of actual time constraints or because of inclusion in lengthier questionnaires” (Marwit & Meuser, 2005). Therefore, the manufacturers of the MM-CGI developed the short form of the initial inventory. The short form of the inventory is also a self-report questionnaire and includes only 18 items out of the 50 items of the full version of the MM-CGI. Those items, which arose through an intercorrelation procedure, were the items best identified to each factor (subscale) and are significantly related to the MM-CGI subscales. Thus, the factorial structure of the MM-CGI-SF remained the same, with the same three factors (subscales) (Factor 1: Personal Sacrifice Burden, Factor 2: Heartfelt Sadness and Longing, Factor 3: Worry and Felt Isolation). Each factor (subscale) of the MM-CGI-SF has six items. In respect of internal consistency, the items of each of the three subscales of the MM-CGI-SF showed good reliability coefficients (Cronbach’s *α* of 0.83, 0.80, and 0.80, respectively, for each subscale) (Marwit & Meuser, 2005).

Concerning the relationship between the MM-CGI and MM-CGI-SF, the examination of the concurrent validity of the short form of the inventory showed that the three factors (subscales) of the MM-CGI-SF were strongly correlated with the three factors (subscales) of the MM-CGI (Marwit & Meuser, 2005).

The psychometric properties of the MM-CGI and MM-CGI-SF have already been examined in other samples and different cultural contexts. Specifically, studies examining the psychometric properties of the MM-CGI and/or MM-CGI-SF have been conducted in Polish (Warchol-Biedermann et al., 2014), Asian and multiethnic Asian (W. C. H. Chan et al., 2017; I. Chan et al., 2020; Li et al., 2021; Liew et al., 2018a, 2018b), Puerto Rican (Alvelo et al., 2018), Spanish (Sánchez-Alcón et al., 2024), UK (Gilsenan et al., 2022), Turkish (Ar Karci & Karanci, 2020), and Brazilian (Mattos et al., 2023) populations of caregivers. As a result, Polish, Chinese, Mandarin-Chinese, Spanish, Turkish, and Portuguese versions exist.

Regarding evidence of the convergent/divergent and/or discriminant validity of the MM-CGI, during its development, the inventory was administered and tested with the following tools: the Beck Depression Inventory (BDI), Anticipatory Grief Scale (AGS), Geriatric Depression Scale (GDS), Caregiver Strain Index (CSI), Caregiver Well-Being Scale—Basic Needs Subscale (CWBS-BN), Perceived Social Support Questionnaire—Family Subscale (PSSQ-FA), and Clinical Dementia Rating Scale (CDR) (Marwit & Meuser, 2002). All aforementioned tools were completed by family caregivers of persons with dementia. The majority of the tools had statistically significant high correlations with the total score of the MM-CGI (*r* = 0.758 with BDI, *r* = 0.714 with GDS, *r* = 0.798 with AGS, *r* = 0.656 with CSI, *r* = −0.656 with CWBS-BN, and *r* = −0.360 with PSSQ-FA) (Marwit & Meuser, 2002).

Regarding evidence of the convergent/divergent and/or discriminant validity of the MM-CGI-SF, the following tools were administered: the Marwit–Meuser Caregiver Grief Inventory (MM-CGI), Beck Depression Inventory (BDI), Anticipatory Grief Scale (AGS), Geriatric Depression Scale (GDS), Caregiver Strain Index (CSI), Caregiver Well-Being Scale—Basic Needs Subscale (CWBS-BN), and Perceived Social Support Questionnaire—Family Subscale (PSSQ-FA) (Marwit & Meuser, 2005). The majority of the tools also had statistically significant high correlations with the total score of the MM-CGI-SF (*r* = 0.711 with BDI, *r* = 0.689 with GDS, *r* = 0.760 with AGS, *r* = 0.640 with CSI, *r* = −0.592 with CWBS-BN, and *r* = −0.353 with PSSQ-FA) (Marwit & Meuser, 2005).

A noteworthy finding of the constructors of the MM-CGI and MM-CGI-SF concerns the correlations of certain factors with the inventories of strain and of depression. In particular, Factor 1 of the MM-CGI was highly correlated with the CSI (*r* = 0.730), more highly than the other two factors (Marwit & Meuser, 2002). This result indicates the correlation of the strain of the caregivers with the sense of burden in their experience of grief. Additionally, Factor 2 of the MM-CGI showed the lowest correlation with the BDI (*r* = 0.588) and the GDS (*r* = 0.487) compared to the other two factors (Marwit & Meuser, 2002), posing the question of possible differentiation between grief and depression. In accord with the full version of the MM-CGI, the MM-CGI-SF factor correlations showed similar results. That is, Factor 1 of the MM-CGI-SF was highly correlated with the CSI (*r* = 0.680), more highly than the other two factors (Marwit & Meuser, 2005), and Factor 2 of the MM-CGI-SF showed the lowest correlation with the BDI (*r* = 0.503) and the GDS (*r* = 0.390) compared to the other two factors (Marwit & Meuser, 2005).

### Aims of the Study

The endeavor to obtain a reliable and valid tool to assess “anticipatory grief” is important considering the absence, to our present knowledge, of a test of the psychometric properties of a tool which measures the grief of caregivers of persons with dementia in the Greek population. Furthermore, the adaptation of the MM-CGI and the MM-CGI-SF in the Greek population could enhance the early assessment of high levels of grief among Greek family caregivers, in order to potentiate the prevention of depression, burden, and complicated grief in this population.

As noted above, to date, according to our existing knowledge, there is no prior study on the examination of psychometric properties of the MM-CGI and MM-CGI-SF in the Greek population of family caregivers of persons with dementia before death. Thus, this study aims to investigate certain of the psychometric properties (i.e., internal consistency reliability, structural validity, convergent/divergent validity) of the MM-CGI and MM-CGI-SF for the purpose of assessing grief of family caregivers in the Greek population with a reliable and valid tool.

Specifically, the aims of the present study were: (a) the test/confirmation of the three-factor structure of the Greek version of the MM-CGI and MM-CGI-SF, respectively, (b) the evaluation of the internal consistency reliability of the Greek version of the MM-CGI and MM-CGI-SF, respectively, as well as (c) the test of the convergent/divergent and discriminant validity of the Greek version of the MM-CGI and MM-CGI-SF, respectively. In this study, in order to examine the convergent/divergent validity of the MM-CGI and MM-CGI-SF, regarding their assessment of burden and depression, the Zarit Burden Interview (ZBI) and Beck Depression Inventory II (BDI-II) were administered, respectively, and, in addition, the Mini-Mental State Examination (MMSE) was administered in order to explore the relationship between the MM-CGI/MM-CGI-SF (discriminant validity) and the cognitive status of the person with dementia. Specifically, based on the findings of the constructors of the MM-CGI and MM-CGI-SF, we expected that the ZBI would exhibit the highest correlation with Factor 1 (Personal Sacrifice Burden) of the Greek version of the MM-CGI and MM-CGI-SF, while the BDI-II would exhibit the lowest correlation with Factor 2 (Heartfelt Sadness and Longing) of the Greek version of the MM-CGI and MM-CGI-SF. As there were no previous research findings regarding the MMSE, we aimed to investigate whether the cognitive status of the person with dementia, which the MMSE measures, has a potential correlation with the Greek versions of the MM-CGI and MM-CGI-SF, which assess the grief of family caregivers.

## 2. Methods

### 2.1. Participants and Procedure

The sample included five hundred and fifteen (515) family caregivers (226 spouses and 289 offspring) of patients with dementia. The majority of the sample was female (162 males and 353 females), with a mean age of 59.9 years (SD = 14.84). A number of participants (113 family caregivers) were recruited from the Department of Caregiver’s Support of the day care center of the Greek Association of Alzheimer’s Disease and Related Disorders (GAADRD) in Thessaloniki, which is a town in northern Greece. Additionally, a number of family caregivers (402) were recruited from all over Greece (via a Google Form invitation to participate in research about a new inventory that estimates the grief of family caregivers of patients with dementia). Circulation and follow-up of the aforementioned invitation—with the resulting random data collection from all over Greece—was conducted by students from the University of Ioannina in Greece during their attendance and research exercises of two introductory courses in psychology. In cases where the completion of the questionnaires took place in the presence of the researcher (staff of day care center or student), assistance or clarifications were provided where necessary. Participants were encouraged to respond honestly to ensure the reliability of the result.

It should be noted that there were no separate administrations of the MM-CGI and the MM-CGI-SF. The participants completed only the Marwit–Meuser Caregiver Grief Inventory, as the items of the MM-CGI-SF are included in the MM-CGI extended form. Regarding the patients whose caregivers participated in the study, all types of diagnosis of dementia were accepted, and the stages encompassed mild, moderate, and severe dementia and the post-death stage. More details about the sociodemographic and caregiving characteristics of the sample are presented in Table 1.

### 2.2. Ethical Considerations

First of all, the permission of the creators of the inventories MM-CGI and MM-CGI-SF was asked. Furthermore, regarding personal data, the ethical rights of the participants were respected, and the research was carried out in compliance with the relevant European Union law, namely the General Data Protection Regulation (GDPR), which has been in effect since 25 May 2018. According to the law, the use of sensitive personal data is allowed only due to research reasons. The study’s protocol followed the principles outlined in the Helsinki Declaration and was approved by the Scientific Committee of Bioethics and Deontology of the GAADRD (scientific committee approval meeting number: 68/15/5/2021).

### 2.3. Instruments

#### 2.3.1. Marwit–Meuser Caregiver Grief Inventory (MM-CGI)

The MM-CGI was created by Marwit and Meuser in 2002 (Marwit & Meuser, 2002) in order to measure the reactions of grief of the family caregivers of patients with dementia, both spouses and offsprings. The inventory is based on the model of grief of family caregivers and of clinical assessment created by Marwit and Meuser in 2001 (Meuser & Marwit, 2001). The tool consists of 50 items, and the participant is requested to complete the grade of disagreement or agreement on a Likert scale from 1 (strongly disagree) to 5 (strongly agree). The MM-CGI consists of three subscales/factors and a total score of grief (TG). Subscale/Factor 1, called Personal Sacrifice Burden, contains 18 items and measures the experience of personal losses of the caregiver as a result of caregiving, i.e., what they had to give up in order to be a caregiver. Subscale/Factor 2, called Heartfelt Sadness and Longing, contains 15 items and measures the intrapersonal emotional reactions of the caregiver in response to caregiving, and more particularly the emotional reaction to loss of the patient who receives care. Subscale/Factor 3, called Worry and Felt Isolation, contains 17 items and measures the feelings of loss of socializing and support from others. The score of total grief is the sum of the scores of each sentence completed with the grade of agreement or disagreement. For the sentences 8, 22, 23, 45, and 47, which have a negative wording, the score must be reversed before the sum for the total score. A high score on total grief indicates high levels of grief.

#### 2.3.2. Marwit–Meuser Caregiver Grief Inventory—Short Form (MM-CGI-SF)

The MM-CGI-SF is the short form of the MM-CGI. The MM-CGI-SF derives from the MM-CGI and has been found to maintain the same subscales/factors and similar psychometric properties as the initial form of 50 items. It is based on an intercorrelation procedure of a study by Marwit and Meuser (Marwit & Meuser, 2005). Thus, it includes the same three subscales as the MM-CGI version, and it consists of 18 items, 6 items per subscale/factor. Subscale/Factor 1 contains the items 1, 3, 29, 39, 41, and 42 of the full-version MM-CGI. Subscale/Factor 2 contains the items 9, 18, 19, 30, 31, and 38, and Subscale/Factor 3 contains the items 5, 12, 13, 16, 33, and 34 of the MM-CGI. The scoring procedure for the participant is the same as in the MM-CGI, with the difference that there are no reverse score items. A high score on total grief indicates high levels of grief.

#### 2.3.3. Development of the Greek Version of the Marwit–Meuser Caregiver Grief Inventory

In brief, the methods included forward translation, back-translation, and a debriefing to a pre-testing sample of caregivers (Beaton et al., 2000). The steps are described below.

In the first method (forward translation), two psychologists who are native speakers of the Greek language, and have an excellent knowledge of the English language, translated the MM-CGI into Greek versions independently. The two Greek versions were discussed by the psychologists, and one reconciled version was finally reviewed by a professor of English literature for grammatical and syntactical structure and for a more elegant translation of the original text.

In the second case (back-translation), a native speaker of the English language, with excellent knowledge of the Greek language, back-translated the Greek version of the MM-CGI without having seen its original English version. Then, the back-translated version was compared with the original translation to test for any discrepancies, and we reached the final Greek version of the MM-CGI. The professional translator confirmed that the Greek version of the MM-CGI is an exceptionally accurate translation into Greek, with exact renderings of idioms, emotions, and technical terms.

The final step included cognitive debriefing with a pre-testing sample of family caregivers who speak Greek, with qualitative interviews of small groups of people. In this way, we checked the level of comprehensibility and cognitive equivalence of the translation. We conducted cognitive debriefing with 20 family caregivers of each type and severity of dementia. During the cognitive debriefing, we asked the caregivers to tell us their thoughts about the issue that the inventory assesses (using the question, “What do you think the inventory is supposed to assess?”). We also asked caregivers to express their difficulties in understanding any item-question of the inventory. The pre-testing phase of the Greek version of the MM-CGI showed that caregivers adequately understand the contents of the inventory and the subscales (they responded that the items assess the burden, the depression, and the feelings of caregivers, along with sentiments of loss and changes in the everyday life of the caregiver, all of which are components of the grief of caregivers), and they did not have any particular difficulties.

#### 2.3.4. Zarit Burden Interview (ZBI)

The ZBI was developed by Zarit and his colleagues and measures the caregiver’s perceived physical, emotional, and social burden during the dementia progression (Zarit et al., 1985). In particular, it consists of 22 items scored from 0 (never) to 4 (nearly always). A higher total score means a higher level of perceived burden. The Greek translation and adaptation of the ZBI was made by Papastavrou et al. (2007) and showed satisfactory psychometric properties. In the present study, the Cronbach’s *α* of the ZBI was excellent (*α* = 0.92).

#### 2.3.5. Beck Depression Inventory II (BDI-II)

The BDI-II was originally developed by Beck and his colleagues and is widely used in order to assess cognitive, emotional, behavioral, and physical symptoms of depression (Beck et al., 1988). The BDI-II includes 21 items (scored from 0 to 3), and higher total scores indicate higher levels of severity of depressive symptoms. The Greek version of the BDI-II showed very good psychometric properties regarding validity and reliability in the Greek population (Cronbach’s *α* = 0.93 and test–retest reliability with a Pearson coefficient between 0.75 and 0.98) (Fountoulakis et al., 2003). In the present study, the Cronbach’s *α* of the BDI-II was excellent (*α* = 0.92).

#### 2.3.6. Mini-Mental State Examination (MMSE)

The MMSE was created by Folstein et al. (1975) and is a tool used to evaluate cognitive status (orientation to time and place, registration, attention and calculation, recall, language, and visuo-construction ability). It is a 30-point test/examination, with higher scores indicating better cognitive performance and lower scores indicating severe cognitive decline. The validation in the Greek population was done by Greek researchers (Fountoulakis et al., 2000), and the tool appeared to be valid during testing and retesting, with a Spearman’s coefficient of *p* = 0.98 (*p* < 0.001). According to the score level of 23/24 in the Greek population, sensitivity is 90.80, specificity 90.62, and positive predictive value 92.94 (Fountoulakis et al., 2000).

### 2.4. Data Analysis

Although exploratory factor analysis (EFA) is useful in test construction, it does not provide an especially convincing test of the factorial structure of an inventory, as it does not permit the investigator to hypothesize and confirm which of a series of alternative plausible latent factor models best fits the data. Confirmatory factor analysis (CFA) is known to provide more detailed and sophisticated information regarding factor structure of the previously validated scales (Kelloway, 1995; Kline, 2005). Thus, CFA was preferred over EFA in the current study to test the construct validity of the Greek version of the Marwit–Meuser Caregiver Grief Inventory (both the MM-CGI and MM-CGI-SF).

Structural equation models were conducted in the statistical program EQS 6.1. (Bentler, 2005) and were performed on the covariance matrices of the 50 items and the 18 items, respectively, of each form of the inventory, using the maximum-likelihood estimation procedure. The Wald test and the Lagrange multiplier test (LMT) were used to suggest more restricted models. A non-statistical significance of the *χ*^2^-test indicates that the implied theoretical model significantly reproduces the sample variance–covariance relationships in the matrix. As this test is sensitive to sample size, model fit was also evaluated by using the root mean squared error of approximation (RMSEA). The RMSEA tests how well the model would fit the population covariance matrix. A rule of thumb is that RMSEA ≤ 0.06 indicates close approximate fit. The Comparative Fit Index (CFI), which is one of the indices assessing the relative improvement in fit of the researcher’s model compared with a baseline model, was also used. A rule of thumb for the CFI is that values close to 0.95 or greater may indicate reasonably good fit of the researcher’s model. In addition, model fit was evaluated by using the standardized root mean squared residual (SRMR). The SRMR is a measure of the mean absolute correlation residual, the overall difference between the observed and the predicted correlations. Values of the SRMR less than 0.08 are generally considered favorable (Brown, 2006; Hu & Bentler, 1999; Kline, 2005).

As regards the sample size requirements, for SEM techniques, it is recommended as a rule of thumb that there be at least five observations per estimated variable (Hair et al., 1998). A total of 50 and 18 variables, respectively, were estimated in confirmatory factor models. Hence, the sample size for the CFA models had to exceed 250. Thus, the sample size exceeded the minimum recommended level for performing confirmatory factor analyses.

The rest of the statistical analyses—except for CFA—which include calculation of Cronbach’s *α* internal consistency reliability coefficients and Pearson’s *r* correlation coefficients, were performed with the use of IBM SPSS Statistics version 25, and the statistical significance was set at 0.05.

## 3. Results

### 3.1. Test of MM-CGI and MM-CGI-SF Factor Structure

To evaluate the three-factor structure—namely, Factor 1: Personal Sacrifice Burden, Factor 2: Heartfelt Sadness and Longing, and Factor 3: Worry and Felt Isolation—of the Greek version of the Marwit–Meuser Caregiver Grief Inventory (MM-CGI), a set of confirmatory factor analyses was conducted of the data collected from the 50 items that constitute it. CFA began with the examination of the measurement model, with which the three latent variables (factors) showed no correlations (interrelations). The measurement model (Model MA.1) was not acceptable according to the indices of fit: *χ*^2^(1175, *N* = 517) = 5960.81, *p* < 0.000, CFI = 0.65, SRMR = 0.27, RMSEA = 0.09 (CI90% 0.08–0.09) (Brown, 2006; Hu & Bentler, 1999; Kline, 2005). Nevertheless, all parameters were found to be statistically significant in this model, as none of them was dropped in the process of the Wald test.

Then, the structural model was tested, according to which there were interrelations among the three factors. At this second performance of CFA, latent variables (factors) were allowed to freely intercorrelate. However, during our trial to test the structural model, the EQS program (Bentler, 2005) warned that “test results may not be appropriate due to condition code”. As it was obvious that the addition of the interrelations among the three factors was the cause of condition code, we decided to test an alternative three-factor model in which the three factors/subscales—namely, Personal Sacrifice Burden, Heartfelt Sadness and Longing, and Worry and Felt Isolation—were first-order latent variables (factors) that loaded on a second-order latent factor called MM-CGI-Total.

However, during our trial to test the aforementioned model, the EQS program (Bentler, 2005) warned that a parameter estimate is not inside the specific boundaries. Therefore, we proceeded with the identification of the alternative model’s parameter that contributed most to the misfit. More specifically, the disturbance of the first-order Worry and Felt Isolation factor was being held at the lower boundary (0.000) specified for the problem. The constraint of this parameter at a lower boundary indicates a solution which is not acceptable: that the first-order Worry and Felt Isolation factor could be perfectly predicted from the second-order latent factor called MM-CGI-Total.

Therefore, we decided to slightly modify the alternative three-factor model to a two-factor model in which the two factors/subscales—namely Personal Sacrifice Burden, and Heartfelt Sadness and Longing—were first-order latent variables (factors), and both these first-order factors as well as all the remaining 17 measured variables (items)—that were not included in them, as they came from the Worry and Felt Isolation subscale according to the MM-CGI creators—were loaded on a second-order latent factor called MM-CGI-Total.

This slight modification allowed the alternative model to run, and the conduction of the residual analysis, the Wald test, and the Lagrange multiplier test improved the fit of the final model (Model MA.2) on all indices, except for the comparative fit index (CFI), whose value fell at the lowest boundary of the marginal range of 0.90–0.95 and was indicative of marginally acceptable model fit (Brown, 2006): *χ*^2^(1041, *N* = 512) = 2450.01, *p* < 0.000, CFI = 0.90 (0.897), SRMR = 0.05, RMSEA = 0.05 (CI90% 0.05–0.06), as a rule of thumb for the CFI is that values greater than 0.90 may indicate reasonably good fit of the researcher’s model (Kline, 2005).

**Figure 1:**
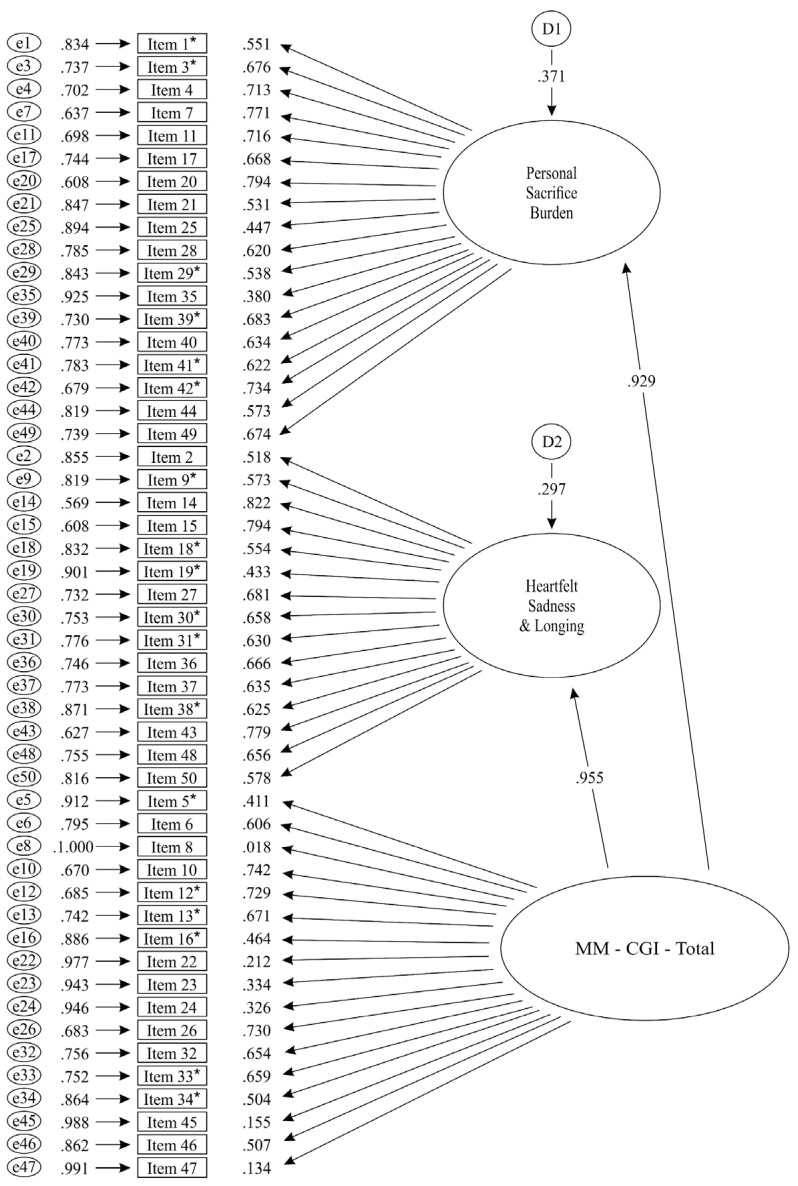
Model MA.2 for the structure of the Greek version of the Marwit–Meuser Caregiver Grief Inventory (MM-CGI) in the test sample (standardized solution).

For each confirmatory factor analysis (CFA) model that was tested, a single path was freed from the relevant latent variable (factor) to each measured variable (item). No cross-loadings were allowed. In the last marginally confirmed model, the first-order latent variables (factors) were defined without any covariance (interrelation) between them, as they loaded on a second-order latent factor. For this model, the metric was set by fixing factor variances to 1.00, except for the second-order latent factor variance, which was fixed to 0.60. Specifically, the final model, which was marginally verified, was a two-factor model in which the two factors/subscales—namely Personal Sacrifice Burden, and Heartfelt Sadness and Longing—were first-order latent variables (factors). Both these first-order factors and all the other 17 measured variables (items)—which were not included in them, as they came from the Worry and Felt Isolation subscale according to the MM-CGI creators—loaded on a second-order latent factor called MM-CGI-Total. This second-order factor was found to be identical to the third subscale of the MM-CGI—namely, Worry and Felt Isolation—and it simultaneously included the two first-order factors (latent variables), which represented the other two subscales of the MM-CGI. The factor structure of the Greek version of the Marwit–Meuser Caregiver Grief Inventory (MM-CGI), as it was finally confirmed in Model MA.2, is presented in Figure 1.

***Note 1:*** D1 = Error variance of first-order latent variable/factor 1 (Sacrifice Burden); D2 = Error variance of first-order latent variable/factor 2 (Heartfelt Sadness and Longing); e1 to e50 = Error variances of measured variables 1 to 50.
***Note 2:*** All loadings and parameters presented are statistically significant (*p* < 0.05) except for the measured variable MM-CGI Item 8’s loading on the second-order factor MM-CGI-Total (*p* = 0.70).
***Note 3:*** The 18 items of the MM-CGI-SF, which are also included in the MM-CGI, are indicated with (*).

Consequently, considering that CFA marginally confirmed a slightly different factor structure than the one suggested for the MM-CGI by its constructors, a second set of confirmatory factor analyses was performed, using a revised item set of the three MM-CGI subscales. In this revised item set, we excluded 32 items, which, based on an intercorrelation procedure of a study by Marwit and Meuser (2005), were proposed as poorly performing, in order to conclude the MM-CGI-SF, which is the short form of the MM-CGI. It should be noted that the 18 items of the MM-CGI-SF derive directly from the MM-CGI and have been found to maintain the same subscales/factors as the initial form of 50 items.

Therefore, to test the three-factor structure—namely, Factor 1: Personal Sacrifice Burden, Factor 2: Heartfelt Sadness and Longing, and Factor 3: Worry and Felt Isolation—of the Greek version of the Marwit–Meuser Caregiver Grief Inventory—Short Form, the second set of confirmatory factor analyses was conducted on the data collected only from the 18 items of the MM-CGI, which are also those that constitute the MM-CGI-SF. Specifically, the MM-CGI-SF consists of 18 items, with six items per subscale/factor. Subscale/Factor 1 contains the items 1, 3, 29, 39, 41, and 42 of the full-version MM-CGI. Subscale/Factor 2 contains the items 9, 18, 19, 30, 31, and 38, and Subscale/Factor 3 contains the items 5, 12, 13, 16, 33, and 34 of the MM-CGI.

The conduction of the confirmatory factor analyses began with the examination of the measurement model, with which the three latent variables (factors) showed no correlations (interrelations) among them. The measurement model (Model MB.1) was not acceptable according to various indices of fit: *χ*^2^(135, *N* = 512) = 1169.69, *p* < 0.000, CFI = 0.70, SRMR = 0.25, RMSEA = 0.12 (CI90% 0.12–0.13) (Brown, 2006; Hu & Bentler, 1999; Kline, 2005). Nevertheless, all parameters were found to be statistically significant in this model, as none of them was dropped in the process of the Wald test.

Then, the structural model (Model MB.2) was tested, according to which there were interrelations among the three factors. Although the structural model was not acceptable, the indices in this model were improved compared to the measurement model: *χ*^2^(132, *N* = 512) = 640.23, *p* < 0.000, CFI = 0.85, SRMR = 0.06, RMSEA = 0.09 (CI90% 0.08–0.09) (Brown, 2006; Hu & Bentler, 1999; Kline, 2005). Furthermore, all parameters were found to be statistically significant in this model too, since none of them was dropped in the process of the Wald test.

**Figure 2:**
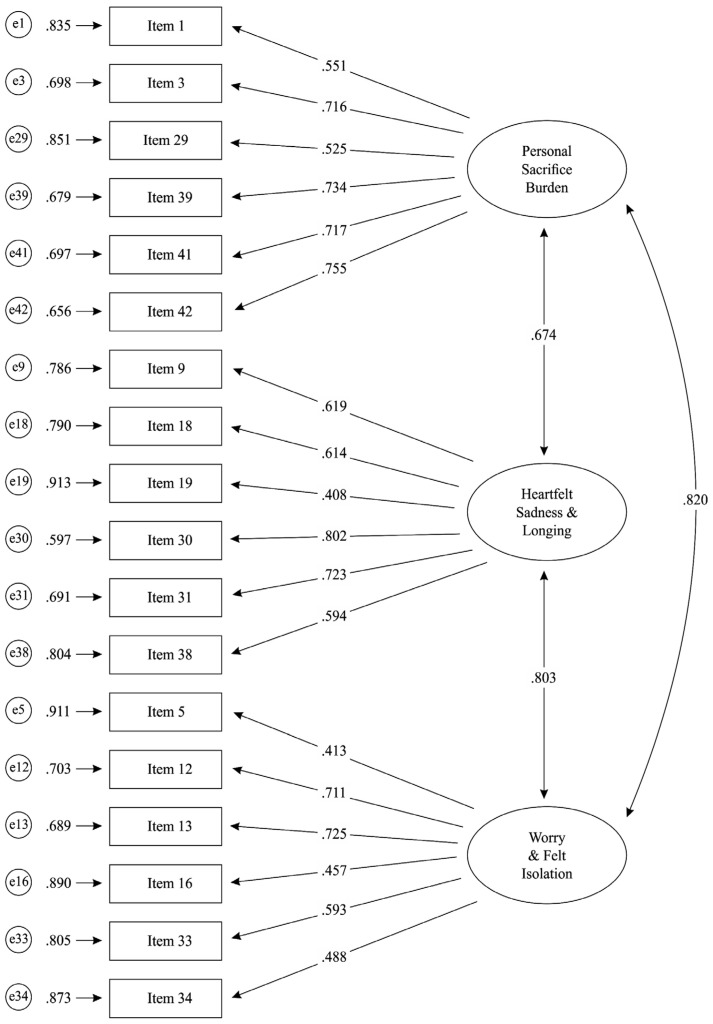
Model MB.3 for the structure of the Greek version of the Marwit–Meuser Caregiver Grief Inventory—Short Form (MM-CGI-SF) in the test sample (standardized solution).

Therefore, we proceeded with the identification of the areas of the structural model that contributed most to the misfit. Residual analysis was conducted, and the Wald test and the Lagrange multiplier test were performed. Different models were tested, and the modifications indicated by the above-mentioned tests were included in the model being tested each time. The modifications were correlated errors of measured variables (items) that were manifested as large standardized residuals. The modifications are presented in the notes of Figure 2 and were covariances (correlations) between errors of indicators (items), which were mainly similarly worded (for example, Item 16: “My friends simply don’t understand what I’m going through”, and Item 34: “The people closest to me do not understand what I’m going through”), or differentially prone to social desirability (for example, Item 38: “I’ve lost other people close to me, but the losses I’m experiencing now are much more troubling”, and Item 39: “Independence is what I’ve lost…I don’t have the freedom to go and do what I want”) (Brown, 2006). Because, according to Brown (2006), in CFA construct validation studies, correlated errors may be needed to account for method covariance, it seems necessary to add some of them in the tested model, as in our construct validation study, we analyze indicators collected from slightly different assessment modalities. Specifically, as we have written in Section 2.1 (Participants and Procedure), the 113 participants were recruited from the Department of Caregiver’s Support of the day care center of the Greek Association of Alzheimer’s Disease and Related Disorders (GAADRD) in Thessaloniki, and the completion of the questionnaires took place in the presence of the researcher (staff of day care center or student), and assistance or clarifications were provided where necessary. On the other hand, there were 402 family caregivers that completed the inventory via a Google Form.

The fit indices of the final structural model (Model MB.3) indicated that this model fits the data sufficiently well [*χ*^2^(115, *Ν* = 512) = 280.69, *p* = 0.000, CFI = 0.95, SRMR = 0.04, RMSEA = 0.05 (CI90% 0.05–0.06)] (Brown, 2006; Hu & Bentler, 1999; Kline, 2005). All parameters were found to be statistically significant in this model too, as none of them was dropped in the process of the Wald test.

For each confirmatory factor analysis (CFA) model that was tested, a single path was freed from the relevant latent variable (factor) to each measured variable (item). No cross-loadings were allowed. In the verified final model (Model MB.3), the three latent variables (first-order factors) were defined with covariances (interrelations) among them. For all the models that were tested in this second set of CFA, the metric was set by fixing latent variable (factor) variances to 1.00.

Specifically, the final model (Model MB.3) fully verified the three-factor structure—namely, Factor 1. Personal Sacrifice Burden, Factor 2: Heartfelt Sadness and Longing, and Factor 3: Worry and Felt Isolation—of the Greek version of the Marwit–Meuser Caregiver Grief Inventory—Short Form (MM-CGI-SF). The three-factor structure of the Greek version of the Marwit–Meuser Caregiver Grief Inventory—Short Form (MM-CGI-SF), as it was finally confirmed in Model MB.3, and the interrelations among the three factors are presented in Figure 2.

***Note 1:*** All loadings and parameters presented indicate statistically significant associations (*p* < 0.05).
***Note 2:*** e = Error variance of measured variable.
***Note 3:*** Covariances (correlations) among errors of measured variables (items) of the Greek version of the Marwit–Meuser Caregiver Grief Inventory–Short Form (MM-CGI-SF) in the test sample (standardized solution): E30 − E3 = −0.168, E16 − E5 = 0.153, E31 − E5 = −0.196, E34 − E5 = 0.270, E42 − E5 = 0.132, E19 − E16 = 0.162, E31 − E16 = −0.098, E34 − E16 = 0.532, E34 − E19 = 0.142, E42 − E19 = 0.099, E31 − E29 = 0.202, E38 − E33 = 0.187, E42 − E33 = 0.230, E39 − E34 = 0.161, E42 − E34 = 0.135, E39 − E38 = 0.279, E42 − E38 = 0.248. All correlations presented indicate statistically significant associations (*p* < 0.05) among errors of measured variables.

Finally, we compared Model MA.2 with Model MB.3 using the chi-square difference test. The results showed that the Δ*χ*^2^ was significant: Model MA.2 and Model MB.3: Δ*χ*^2^(Δ*df* = 926) = 2169.32, *p* < 0.001. Thus, on the basis of the chi-square difference test and comparison of CFI, the best-fitting model was unambiguously the three-factor MM-CGI-SF model with interrelations between the latent variables (factors). Moreover, this model also has the advantage of previous theoretical and empirical validation of the MM-CGI-SF and offers the opportunity to find a score for Factor 3: Worry and Felt Isolation.

### 3.2. Test of MM-CGI-SF Internal Consistency Reliability

Τhe internal consistency reliability of the subscales of the Greek version of the MM-CGI-SF was evaluated with Cronbach’s *α* coefficient. As shown in Table 2, Cronbach’s *α* coefficients of all scales of the MM-CGI-SF were good and acceptable and ranged between 0.79 and 0.90. These findings were closely aligned with the results found by Marwit and Meuser at the development of the MM-CGI-SF (Cronbach’s *α* of 0.83, 0.80, and 0.80, respectively, for each factor) (Marwit & Meuser, 2005). The Cronbach’s *α* internal consistency coefficient of the Greek version of the MM-CGI total grief was also good and acceptable (Cronbach’s *α* of 0.96). This finding is identical with the result found by Marwit and Meuser at the development of the MM-CGI (Cronbach’s *α* of 0.96 for the total inventory) (Marwit & Meuser, 2002).

### 3.3. Test of MM-CGI-SF Internal Validity

The three subscales and the total score of the Greek version of the MM-CGI-SF showed positive and statistically significant correlations at the <0.01 level, two-tailed. A full correlation matrix among measures is presented in Table 3.

### 3.4. Test of MM-CGI-SF Convergent Validity

To examine the convergent and discriminant validity of each of the three subscales and the total score of the Greek version of the MM-CGI-SF with the Zarit Burden Interview (ZBI), the Beck Depression Inventory II (BDI-II), and the Mini-Mental State Examination (MMSE), the Pearson correlation coefficient between them was calculated. The variables were created based on the totals of the ZBI, BDI-II, and MMSE.

The application of correlation analysis revealed statistically significant correlations between the totals of the ZBI, BDI-II, MMSE, and all scales of the MM-CGI-SF, as shown in Table 3.

In particular, the application of the Pearson analysis revealed moderate to high positive statistically significant correlations between the ZBI total score and the four scales of the MM-CGI-SF (ΜΜ-CGI-SF Factor 1 *r* = 0.727, ΜΜ-CGI-SF Factor 2 *r* = 0.579, ΜΜ-CGI-SF Factor 3 *r* = 0.615, ΜΜ-CGI-SF total grief *r* = 0.736).

Furthermore, the application of the Pearson analysis revealed moderate positive statistically significant correlations between the BDI-II total score and the four scales of the MM-CGI-SF (ΜΜ-CGI-SF Factor 1 *r* = 0.447, ΜΜ-CGI-SF Factor 2 *r* = 0.429, ΜΜ-CGI-SF Factor 3 *r* = 0.538, ΜΜ-CGI-SF total grief *r* = 0.548).

However, low to moderate negative statistically significant correlations emerged between the MMSE test and the four scales of the MM-CGI-SF (ΜΜ-CGI-SF Factor 1 *r* = −0.377, ΜΜ-CGI-SF Factor 2 *r* = −0.303, ΜΜ-CGI-SF Factor 3 *r* = −0.237, ΜΜ-CGI-SF total grief *r* = −0.344).

According to the results, convergent and divergent validity are satisfactory for the Greek version of the MM-CGI-SF and the Greek population. A full correlation matrix among measures is presented in Table 3.

## 4. Discussion

The present study was conducted to broaden the knowledge on the assessment of the grief of family caregivers of persons with dementia by examining the psychometric properties of the MM-CGI and the MM-CGI-SF in the Greek population. The attempt to examine an inventory specific to the grief of family caregivers of persons with dementia is important considering the existing lack of such a tool in the Greek literature.

According to the investigation of structural validity in other countries’ samples, the first Spanish-language version of the MM-CGI-SF was found not to be equivalent with regard to the dimensionality of the original inventory. The first Spanish-language version on a Puerto Rican sample of 100 caregivers resulted in a one-factor solution which had a better fit with their research data than the original three-factor model (Alvelo et al., 2018). However, the second Spanish-language version of the MM-CGI-SF was conducted with a Spanish population, from Spain, on a sample of 250 caregivers, and a culturally different sample of Puerto Ricans, and showed the same three factors (subscales) and variables (items) as the original inventory, via confirmatory factor analysis (CFA) (Sánchez-Alcón et al., 2024).

The structure of a Chinese version of the MM-CGI and MM-CGI-SF was examined, via confirmatory factor analysis and exploratory factor analysis (EFA), in a multiethnic Asian population of 300 caregivers (Liew et al., 2018a). In the CFA, the original three-factor model of the MM-CGI showed modest model fit, while the fit indices of the three-factor MM-CGI-SF showed better model fit. The test of the structural validity of the Mandarin-Chinese version of the MM-CGI-SF verified the three-factor structure, as in the original version, via an EFA of a sample of 91 caregivers (Li et al., 2021).

A study in a UK sample of 508 caregivers, via EFA, demonstrated that the factor model of MM-CGI was not adequately fitted. On the other hand, the MM-CGI-SF three-factor model showed adequate model fit (Gilsenan et al., 2022). Finally, the Turkish version of the MM-CGI-SF, which had been examined with CFA, showed a good fit to the original three-factor inventory on a sample of 190 caregivers (Ar Karci & Karanci, 2020).

The findings of good internal consistency reliability coefficients of the MM-CGI and MM-CGI-SF corroborate those reported for the Polish version of the MM-CGI (Cronbach’s α coefficient of 0.90) (Warchol-Biedermann et al., 2014), the first Spanish-language version of MM-CGI-SF on a Puerto Rican sample (Cronbach’s α coefficient of 0.91) (Alvelo et al., 2018), the second Spanish-language version of the MM-CGI-SF on a Spanish sample (Cronbach’s α coefficient of 0.85, 0.84, and 0.82, respectively, for Factor 1, Factor 2, and Factor 3 and of 0.93 for total score) (Sánchez-Alcón et al., 2024), the first Chinese version of the MM-CGI-SF (Cronbach’s α coefficient of 0.94 for total score) (W. C. H. Chan et al., 2017), the Mandarin-Chinese version of the MM-CGI (Cronbach’s α coefficient of 0.96) (Liew et al., 2018a), the multiethnic Asian population (Cronbach’s α coefficient of 0.98 for MM-CGI and Cronbach’s α coefficient of 0.94 for MM-CGI-SF) (Liew et al., 2018b), another Mandarin-Chinese version of MM-CGI-SF (Cronbach’s α coefficient of 0.84 for total score) (Li et al., 2021), a study of the MM-CGI-SF with a UK sample (Cronbach’s α coefficients of 0.85, 0.85, and 0.80, respectively, for Factor 1, Factor 2, and Factor 3) (Gilsenan et al., 2022), and finally the Turkish version of the MM-CGI-SF (Cronbach’s α coefficient of 0.88, 0.82, and 0.82, respectively, for Factor 1, Factor 2, and Factor 3 and of 0.92 for total score) (Ar Karci & Karanci, 2020). The cross-cultural adaptation of the MM-CG-SF for the Brazilian population showed similar results of good internal consistency reliability (Cronbach’s α coefficient of 0.84, 0.81, 0.76, respectively, for Factor 1, Factor 2, and Factor 3) (Mattos et al., 2023). In a study of a Mandarin-Chinese version of the MM-CGI in an Asian multiethnic population in Singapore, the researchers, due to poor validation of the original factor structure of the MM-CGI (Liew et al., 2018b), revised the original factor structure and proposed three alternative dimensions with acceptable internal consistency reliability (Cronbach’s α coefficients of 0.95, 0.96, and 0.78, respectively, for Factor 1, Factor 2 and Factor 3) (I. Chan et al., 2020).

In our study, concerning the structural validity of the MM-CGI and MM-CGI-SF, the conduction of CFA did not confirm the structure of the original MM-CGI in the Greek population (Marwit & Meuser, 2002). In particular, the application of confirmatory factor analysis in the Greek version of the MM-CGI marginally confirmed a slightly differentiated structure of the original three-factor model of the MM-CGI. Conversely, when the Greek version of the MM-CGI-SF was tested (via CFA application), its structure was fully verified in reference to the initial structure suggested by the creators of the inventory for its short form (Marwit & Meuser, 2005). These findings are consistent with studies which examined the factorial validity of both the MM-CGI and MM-CGI-SF and found a better fit of the MM-CGI-SF to the original three-factor structure (Liew et al., 2018b; Gilsenan et al., 2022). Additionally, the results of the present study, in regard to the MM-CGI-SF structure, are in line with the investigation of structural validity of the MM-CGI-SF in other countries’ populations of family caregivers. Namely, the second Spanish-language version (Sánchez-Alcón et al., 2024), the Mandarin-Chinese version (Li et al., 2021), and the Turkish version (Ar Karci & Karanci, 2020) of the MM-CGI-SF showed a factorial structure of three factors as the original version, indicating that this type of family caregivers’ pre-death grief is possibly a common cross-cultural phenomenon.

However, the first Spanish-language version of the MM-CGI-SF on a Puerto Rican sample did not confirm the original three-factor model (Alvelo et al., 2018), possibly as a result of the small sample size according to the recommendations of a larger number of cases for the conduction of CFA (Myers et al., 2011).

In respect of the internal consistency reliability, the Cronbach’s *α* coefficients of the Greek version of the MM-CGI-SF indicate a satisfactory internal consistency reliability for the subscales of the inventory, similar to those reported in the initial study by Marwit and Meuser (Marwit & Meuser, 2005). These results are also aligned with other studies examining the internal consistency reliability of this tool (Alvelo et al., 2018; Ar Karci & Karanci, 2020; W. C. H. Chan et al., 2017; Gilsenan et al., 2022; Li et al., 2021; Liew et al., 2018b; Mattos et al., 2023; Sánchez-Alcón et al., 2024).

As to the relationship of the Greek version of the MM-CGI-SF with other psychometric tools, in our study, ZBI total score was found to be strongly positively correlated with Factor 1(Personal Sacrifice Burden) of the MM-CGI-SF, which indicates the convergent character of the attribute of burden of caregiver in the experience of caregiving and grieving. Similar results were found by Meuser and Marwit, with the Caregiver Strain Index (CSI) being the most positively correlated with Factor 1 (Personal Sacrifice Burden) (Marwit & Meuser, 2002, 2005). Consistent results were also observed in studies by W. C. H. Chan et al. (2017) and Sánchez-Alcón et al. (2024), with CSI being most positively correlated with Factor 1 (Personal Sacrifice Burden). In addition, in the study by Alvelo et al. (2018), the MM-CGI-SF was found to have a strong positive correlation with the total of the CSI. Finally, the MM-CGI-SF showed strong positive correlation with the total score of the ZBI in studies by Liew et al. (2018a) and Liew et al. (2018b). ZBI short form (Bédard et al., 2001) correlated the most positively with Factor 1 (Personal Sacrifice Burden) in the research of Gilsenan et al. (2022), as well as ZBI in the research of Ar Karci and Karanci (2020). These findings possibly highlight the burden of the family caregivers and, as regards grief, reflect the personal losses that take place in their life.

Furthermore, in our study, the BDI-II total score showed moderate positive correlations with the four factors of the MM-CGI-SF, the most moderate positive correlation occurring with Factor 2 (Heartfelt Sadness and Longing), a finding which is aligned with the data of Meuser and Marwit (Marwit & Meuser, 2002, 2005) regarding depression symptoms and the sentiments of loss while grieving. Similarly, the research of Ar Karci and Karanci (2020) demonstrated moderate positive correlations of the BDI (Beck et al., 1961) with the total MM-CGI-SF and the three factors (Factor 1: Personal Sacrifice Burden, Factor 2: Heartfelt Sadness and Longing, Factor 3: Worry and Felt Isolation). Furthermore, the data of Alvelo et al. (2018) showed that the total MM-CGI-SF had a positive moderate correlation with the BDI (Beck et al., 1961). This finding could be explained on the basis that grief and clinical depression are concepts with similar characteristics, while also distinguishable. Grief is a normal reaction to a loss, while melancholy and even “depression”—as a condition proposed by the five-stage model of loss of Kübler-Ross (Kübler-Ross & Kessler, 2014)—are normal parts of this process. On the other hand, clinical depression is a severe emotional disorder. The aforementioned differentiation of grief and depression could be useful to clinical practice in order to intervene therapeutically in an appropriate way for each experience.

According to our knowledge of the literature, in the present study, the MMSE was administered and tested for the first time in a study examining the psychometric properties of the MM-CGI-SF. In our study, MMSE total score showed moderate negative correlation with the three subscales and the total MM-CGI-SF, as presented in Table 3. More specifically, lower scores of the MMSE (which suggest more severe cognitive deterioration because of dementia) correlated with higher scores on MM-CGI-SF scales (which suggest greater grief reaction). Anticipatory grief can be experienced through the whole course of the disease. However, it is more present at the moderate and at the severe stage of dementia, because of the slow progressive worsening and the non-curable nature of the disease (Holley & Mast, 2009). Moreover, this result showcases the need for evaluation of pre-death grief, especially in a caregiver of a person with severe dementia, and the MM-CGI-SF may be more applicable to caregivers who care for patients with severe dementia.

All aforementioned psychometric tools (the MM-CGI-SF, ZBI, BDI-II, and MMSE) were found to be related to each other, and the correlation results indicate the good convergent and discriminant validity of the Greek version of the MM-CGI-SF.

There are various benefits from this study. First of all, given that the caregiver’s grief is a vital component of family caregiving in dementia, the assessment of grief with the Greek version of the MM-CGI-SF is demonstrated as important, especially in Greece, where the institution of family is highly established. Specifically, the valid tool of the study is a multidimensional model of grief which offers the opportunity to better recognize the special needs of the caregiver using a grief profile with three aspects. Based on this grief profile, the appropriate intervention can be selected, thus preventing complicated grief and severe clinical depression. Furthermore, it will allow clinicians to detect higher levels of grief in order to intervene early and also assess the effectiveness of interventions in grief with quantitative data. Moreover, the short version of the inventory is applicable to clinical practice, with low administration time in order to avoid over-burdening the caregiver.

In general, we expect that this tool will open roads for further study of the grief of family caregivers in Greece and prevent complicated grief in this population. Another expectation is that more attention will be given to pre-death grief and not only to grief at the post-death stage. Taking care of caregivers, even at the pre-death stage, can result in their coping better with the process of grieving at the post-death stage and adapting to their new life without the role of caregiving, after the biological death of their patient. A way of doing that is through the intervention of pre-death grief groups for caregivers, the effectiveness of which could be evaluated with the Greek version of the MM-CG-SF. Lastly, the inventory could be used as an introductory tool in grief consulting and could support grief work. That is because, from the process of the development of the inventory, during the cognitive debriefing with the pre-testing sample, it was observed that by completing the inventory, the caregiver gained the experience to realize and express feelings and thoughts about the losses related to dementia.

However, the study also has limitations. The major limitation of the present study is that the psychometric properties test of the MM-CGI-SF has been conducted on the MM-CGI-SF items as they are included in the MM-CGI and not after a single administration of the MM-CGI-SF alone in a separate sample. Furthermore, the language of an inventory can have a fundamental influence on individuals’ responses to culturally sensitive questions about grief. The MM-CGI might not include certain items to describe the experience or certain representations of Greek caregivers with respect to grief. Hence, more investigation is needed to test the psychometric properties of both the MM-CGI and MM-CGI-SF, given that this is the first study testing their psychometric properties in a Greek sample. Another limitation is the fact that grief is an extremely personal and unique experience, and there is the possibility that it cannot be fully examined by one questionnaire.

Some prospects for future research include the following. First, studies could include patients with different types of dementia in order to test differences in grief. Similarly, a longitudinal study across time could detect how the experience of grief changes during the stages of dementia. In addition, demographic information of Greek caregivers (e.g., sex and age differences) and the different characteristics of caregiving (e.g., differences between spouses and offspring caregivers, living arrangement, length of caregiving, etc) could be investigated.

In conclusion, although more research is needed to further validate and refine the Greek version of the MM-CGI and the MM-CGI-SF and to replicate our current findings, the results of the present study, given the size of the sample used and the breadth of the variables examined, show that the MM-CGI-SF is a valid and reliable instrument for measuring anticipatory grief and pre-death grief in Greek family caregivers of dementia patients. As family caregivers do not experience only preparatory or anticipatory grief, but also pre-death grief, which means that they grieve real losses that have already come, the Greek version of the MM-CGI-SF could provide an initial base in examining aspects of grief in the Greek cultural context.

## Figures and Tables

**Table 1:** Demographics of participants (*N* = 515).

Variables	N	%	M	SD	Range
Gender	515				
Male	162	31.5			
Female	353	68.5			
Age			59.9	14.84	18–95
Working Status					
Employed	268	52			
Unemployed	110	21.4			
Retired	117	22.7			
Student	20	3.9			
Marital Status					
Married	352	68.1			
Single	77	14.8			
Divorced	36	7.0			
Widowed	33	6.4			
Missing Values	17	3.7			
Type of Relationship					
Spouse	226	43.9			
Offspring	289	56.1			
Living Arrangements					
With Caregiver	276	53.6			
Not With Caregiver	239	46.4			
Stage of Disease					
Mild	125	24.2			
Moderate	128	24.8			
Severe	108	20.8			
Post-Death	96	18.6			
Missing Values	60	11.6			
Length of Caregiving (years)			3.46	3.21	0–24
Anti-Anxiety Medication					
Yes	65	12.6			
No	450	87.4			
Antidepressant Medication					
Yes	51	9.9			
No	464	90.1			
Patient’s Gender					
Male	214	41.5			
Female	300	58.2			
Other	1	0.3			
Participation in Caregiver Support Group					
Yes	112	21.7			
No	403	78.3			

**Table 2:** Internal consistency reliability coefficients for the MM-CGI-SF.

MM-CGI-SF Greek Version	Cronbach’s α
ΜΜ-CGI-SF Factor 1 (Sacrifice Burden).	0.83
ΜΜ-CGI-SF Factor 2 (Heartfelt Sadness and Longing).	0.79
ΜΜ-CGI-SF Factor 3 (Worry and Felt Isolation).	0.79
ΜΜ-CGI-SF total grief	0.90

**Table 3:** Correlations among the Greek version of the MM-CGI-SF, the Zarit Burden Interview (ZBI), the Beck Depression Inventory II (BDI-II), and the Mini-Mental State Examination (MMSE).

	1	2	3	4	5	6	7
1. ΜΜ-CGI-SF Factor 1(Personal Sacrifice Burden)	—						
2. ΜΜ-CGI-SF Factor 2 (Heartfelt Sadness and Longing)	0.580 **	—					
3. ΜΜ-CGI-SF Factor 3 (Worry and Felt Isolation)	0.651 **	0.586 **	—				
4. ΜΜ-CGI-SF total grief	0.868 **	0.840 **	0.868 **	—			
5. ZBI	0.727 **	0.579 **	0.615 **	0.736 **	—		
6. BDI	0.447 **	0.429 **	0.538 **	0.548 **	0.533 **	—	
7. MMSE	−0.377 **	−0.303 **	−0.237 *	−0.344 **	−0.271 *	−0.243 *	—

***Note:*** ** Correlation is significant at the 0.01 level (two-tailed). * Correlation is significant at the 0.05 level (two-tailed).

## Data Availability

The original contributions presented in this study are included in the article. Further inquiries can be directed to the corresponding author.
